# Development of novel monoclonal antibodies against CD109 overexpressed in human pancreatic cancer

**DOI:** 10.18632/oncotarget.25017

**Published:** 2018-04-13

**Authors:** Gustavo A. Arias-Pinilla, Angus G. Dalgleish, Satvinder Mudan, Izhar Bagwan, Anthony J. Walker, Helmout Modjtahedi

**Affiliations:** ^1^ School of Life Sciences, Pharmacy and Chemistry, Kingston University London, Kingston-upon-Thames, Surrey, UK; ^2^ Department of Cellular and Molecular Medicine, St George's University of London, London, UK; ^3^ Department of Surgery of Hammersmith Campus, Imperial College, London, UK; ^4^ Department of Histopathology, Royal Surrey County Hospital, Guildford, UK

**Keywords:** pancreatic cancer, monoclonal antibodies, CD109 antigen, tissue arrays, immunohistochemistry

## Abstract

Pancreatic cancer is one of the most aggressive and lethal types of cancer, and more effective therapeutic agents are urgently needed. Overexpressed cell surface antigens are ideal targets for therapy with monoclonal antibody (mAb)-based drugs, but none have been approved for the treatment of pancreatic cancer. Here, we report development of two novel mouse mAbs, KU42.33C and KU43.13A, against the human pancreatic cancer cell line BxPC-3. Using ELISA, flow cytometry, competitive assay and immunoprecipitation followed by mass spectrometry, we discovered that these two mAbs target two distinct epitopes on the external domain of CD109 that are overexpressed by varying amounts in human pancreatic cancer cell lines. Treatment with these two naked antibodies alone did not affect tumour cell growth or migration *in vitro*. Of the two mAbs, only KU42.33C was useful in determining the expression of CD109 in tumour cells by Western blot and immunohistochemistry. Interestingly, immunohistochemistry of human pancreatic carcinoma tissue arrays with mAb KU42.33C showed that 94% of the 65 human pancreatic adenocarcinoma cases were CD109 positive, with no expression in normal pancreatic tissues. Our results suggest that these two novel mAbs are excellent tools for determining the expression level of CD109 in the tumour specimens and sera of patients with a wide range of cancers, in particular pancreatic cancer, and for investigating its diagnostic, prognostic and predictive value. Further research is warranted and should aim to unravel the therapeutic potential of the humanised forms or conjugated versions of such antibodies in patients whose tumours overexpress CD109 antigen.

## INTRODUCTION

Pancreatic cancer is one of the most aggressive and lethal types of cancer with a five-year survival rate of ∼5% which has barely improved in the past four decades [1]. In 2012, there were an estimated 337,872 new cases and 330,391 deaths as a result of pancreatic cancer worldwide [1]. Treatment of pancreatic cancer requires a multidisciplinary approach that includes surgery, chemotherapy, radiotherapy and palliative care. Surgery is indicated in patients with localised disease, accounting only for 10-20% of cases, and is followed by adjuvant chemotherapy [2]. Treatment of metastatic disease is based on gemcitabine-containing regimens, sometimes in combination with erlotinib or more recently with nab-paclitaxel although such combinations only offer modest survival benefit in some patients in comparison with gemcitabine alone [3–5]. Treatment with gemcitabine-free FOLFIRINOX (leucovorin, fluorouracil, irinotecan, oxaliplatin) has improved the median overall survival compared with gemcitabine alone (11.1 vs 6.8 months), but this combinational therapy is accompanied by increased side effects and thus is often an option for younger patients with good performance status [6]. There is currently no reliable biomarker or screening method for the early detection of pancreatic cancer and novel and more effective therapeutics are urgently needed. Moreover, without further advances in these fronts, pancreatic cancer is projected to bypass breast, prostate and colorectal cancers, and become the second leading cause of cancer deaths after lung cancer in the USA by 2030, and the third leading cause of cancer death in European Union after lung and colorectal cancer by 2025 [7, 8].

Monoclonal antibody (mAb) technology is an excellent tool for the identification of novel and overexpressed cell surface antigens in human malignancies and mAb-based products have untapped therapeutic and diagnostic potential in cancer. Indeed, the exquisite specificity of mAbs for their target antigens makes them an attractive approach for targeted cancer therapy [9–14]. To date, 32 mAbs have been approved for cancer treatment in the U.S. and/or European Union. However, none have yet been approved for pancreatic cancer [15–18]. Our aim was to develop novel mAbs of diagnostic and therapeutic potential against overexpressed cell surface antigens on human pancreatic cancer cells. Here, we report the development of two novel mouse mAbs against distinct epitopes on the extracellular domain of CD109, using the human pancreatic cancer cell line BxPC-3 as the source of tumour immunogen. We showed that the CD109 antigen is overexpressed in several human pancreatic cancer cell lines and pancreatic cancer tissue array, with no expression in normal pancreatic tissues. The characterisation of these antibodies and their potential for use as diagnostic and therapeutic agents are discussed.

## RESULTS

### Development of two novel mouse mAbs KU42.33C and KU43.13A

**Figure 1:**
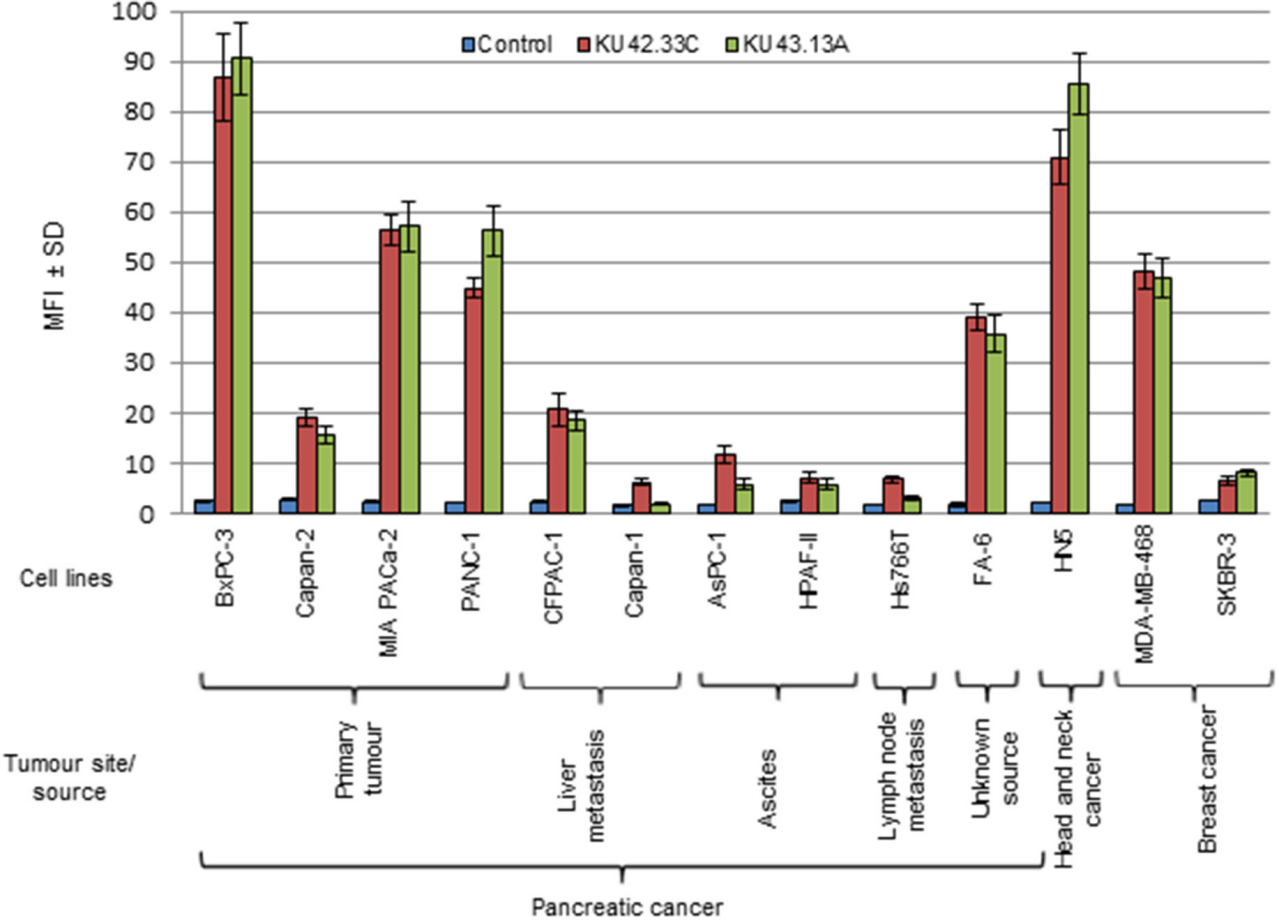
Expression level of the antigens recognised by novel mAbs KU42. 33C and KU43.13A on human pancreatic cancer and other cancer cell lines, determined by flow cytometry Data is presented as Mean Fluorescence Intensity (MFI) ± SD.

Several hybridoma were generated by the fusion of lymphocytes, isolated from the spleens of mice immunised with BxPC-3 cells, and the myeloma cell line SP2. The hybridomas’ supernatants were screened against a panel of human pancreatic cancer cell lines established from patients at different stages of their disease including primary tumours (BxPC-3, Capan-2, MIA PaCa-2, PANC-1), liver metastatic tumours (CFPAC-1, Capan-1), ascites (HPAF-II, AsPC-1) and lymph node metastasis (Hs 766T). The results of ELISA screening showed that the antibodies secreted by two hybridomas, KU42.33C and KU43.13A, were against an antigen with high level of expression in some of the human pancreatic cell lines (e.g. BxPC-3, PANC-1, FA-6 and MIA PaCa-2, Supplementary Figure 1). However, the target antigen recognised by mAbs KU42.33C and KU43.13A was found to be trypsin-sensitive and there was no binding when screened by flow cytometry (data not shown). Therefore, an enzyme-free cell dissociation buffer was used for cell detachment prior to flow cytometry analysis. Following optimisation, high level of expression of the target antigen was found with both mAbs KU42.33C and KU43.13A in some of the human pancreatic cancer cell lines including BxPC-3 (MFI=86 and 90), Capan-2 (MFI=19 and 16), MIA PaCa-2 (MFI=56 and 57), PANC-1 (MFI=45 and 56), CFPAC-1 (MFI=21 and 19) and FA-6 (MFI=39 and 36, respectively), compared to negative control (Figure 1, Supplementary Figure 2). In contrast, the expression of the target antigen in other human pancreatic cancer cell lines namely Capan-1, AsPC-1, HPAF-II and Hs766T was found to be negative or lower with MFI values ranging from 2-12 (Figure 1, Supplementary Figure 2). We also examined the level of expression of the target antigen in other cancer cell lines including human head and neck squamous carcinoma HN5 and breast cancer cell lines MDA-MB-468 and SKBR-3. High level of the target antigen was present in both HN5 and the triple negative breast cancer cell line MDA-MB-468, but not in the HER-2 overexpressing breast cancer cell line SKBR-3 (Figure 1, Supplementary Figure 2).

**Figure 2:**
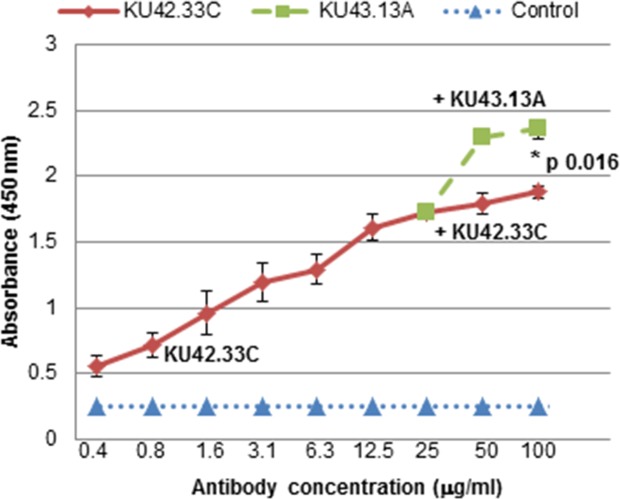
MAbs KU42.33C and KU43.13A are directed against distinct epitopes on BxPC-3 human pancreatic cancer cells BxPC-3 cells were grown in 96-well plates to near confluency. MAb KU42.33C was added in doubling dilutions at highest concentration of 25 μg/ml for 1 hour on ice. After washing unbound antibodies, increasing concentrations of mAb KU42.33C or mAb KU43.13A were added and the total level of bound antibodies were determined as described in Materials and Method section. Data is presented as mean absorbance at 450 nm ± SD.

Both novel mAbs were purified by affinity chromatography and were found to be of IgG1κ isotype (Supplementary Figure 3). To determine whether the two antibodies were directed against the same, overlapping or distinct epitopes on the target antigen, competitive ELISA was performed. For this, saturation of the antigen binding site was initially performed with one the mAbs prior to addition of the second antibody to explore if absorbance value increased further. The absorbance curve for mAb KU42.33C plateaued at concentrations higher than 50 μg/ml. Thereafter, only additional doses of mAb KU43.13A increased the absorbance (Figure 2). These results support that mAbs KU42.33C and KU43.13A recognise non-overlapping epitopes on the extracellular domain on the target antigen.

### The two novel mAbs KU42.33C and KU43.13A are against CD109 on human pancreatic cancer cells

**Figure 3:**
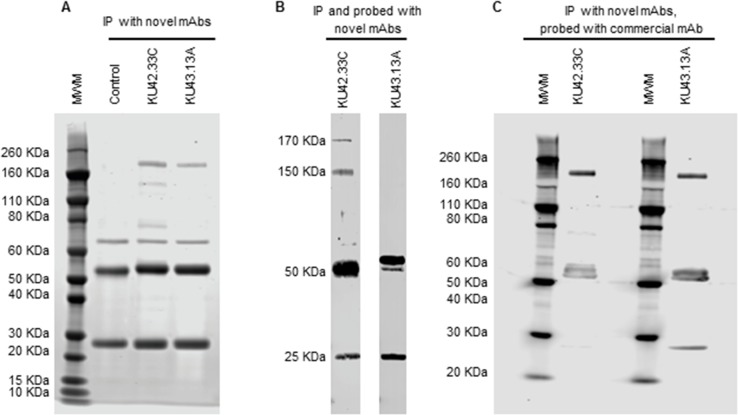
Immunoprecipitation and immunodetection by Western blot of CD109 antigen with novel mAbs KU42.33C and KU43.13A **(A)** Immunoprecipitation was performed with novel mAbs KU42.33C and KU43.13A using sheep anti-mouse dynabeads. A protein band of ∼170 KDa was immunoprecipitated with both novel mAbs. The ∼50/25 KDa bands represent heavy and light chains of the anti-mouse antibody, respectively. **(B)** CD109 antigen was immunoprecipitated and probed with novel mAbs KU42.33C and KU43.13A. CD109 was immunodetected by mAb KU42.33C but not by mAb KU43.13A. **(C)** CD109 antigen was immunoprecipitated with mAbs KU42.33C and KU43.13A and subsequently immunodetected with commercial anti-CD109 mAb. MWM: molecular weight marker.

Both mAbs, KU42.33C and KU43.13A, immunoprecipitated a ∼170 KDa protein identified as CD109 by mass spectrometry (Figure 3A and Table 1). In addition, mAb KU42.33C immunoprecipitated faint bands of ∼150 KDa and ∼80 KDa (Figure 3A). However, in Western blot, only KU42.33C detected the CD109 bands of 170 KDa and 150 KDa, suggesting that KU43.13A is directed against a conformational epitope on CD109 antigen (Figures 3B). Furthermore, to confirm that these two antibodies were directed against CD109, BxPC-3 tumour lysates were immunoprecipitated with the two novel antibodies, transferred by Western blot and then probed with a commercial anti-CD109 antibody. As shown in Figure 3C, the commercial antibody detected the CD109 antigen immunoprecipitated by both of the novel mAbs. These results suggest that mAbs KU42.33C and KU43.13A are directed towards a sequential and a conformational epitope on CD109 antigen, respectively.

**Table 1:** Identification of proteins recognised by novel mAbs KU42.33C and KU43.13A by mass spectrometry

Band No.	mAb	Protein Hits Matches/Sequences		
**1**	KU42.33C	Q6YHK3 CD109 antigen OS=Homo sapiens GN=CD109 PE=1 SV=2 Mass: 162500 Score: 381 Matches: 6(6) Sequences: 6(6)		
		*Start-End*	*Score*	*Peptide*
		375–385	45	K.LSDSWQPR.S
		649–663	48	K.FLIDTHNR.L
		859–872	64	R.ADGNQLTLEER.R
		879–894	99	K.SYSQSILLDLTDNR.L
		1131–1138	53	K.TLSFSFPPNTVTGSER.V
		1220–1227	73	K.DYIDGVYDNAEYAER.F
2	KU43.13A	Q6YHK3 CD109 antigen OS=Homo sapiens GN=CD109 PE=1 SV=2 Mass: 162500 Score: 158 Matches: 3(3) Sequences: 3(3)		
		*Start-End*	*Score*	*Peptide*
		375–385	62	R.ADGNQLTLEER.R
		1131–1138	42	K.LSDSWQPR.S
		1220–1227	54	K.FLIDTHNR.L

### Effect of novel mAbs KU42.33C and KU43.13A on tumour growth and migration and cellular location of CD109

**Figure 4:**
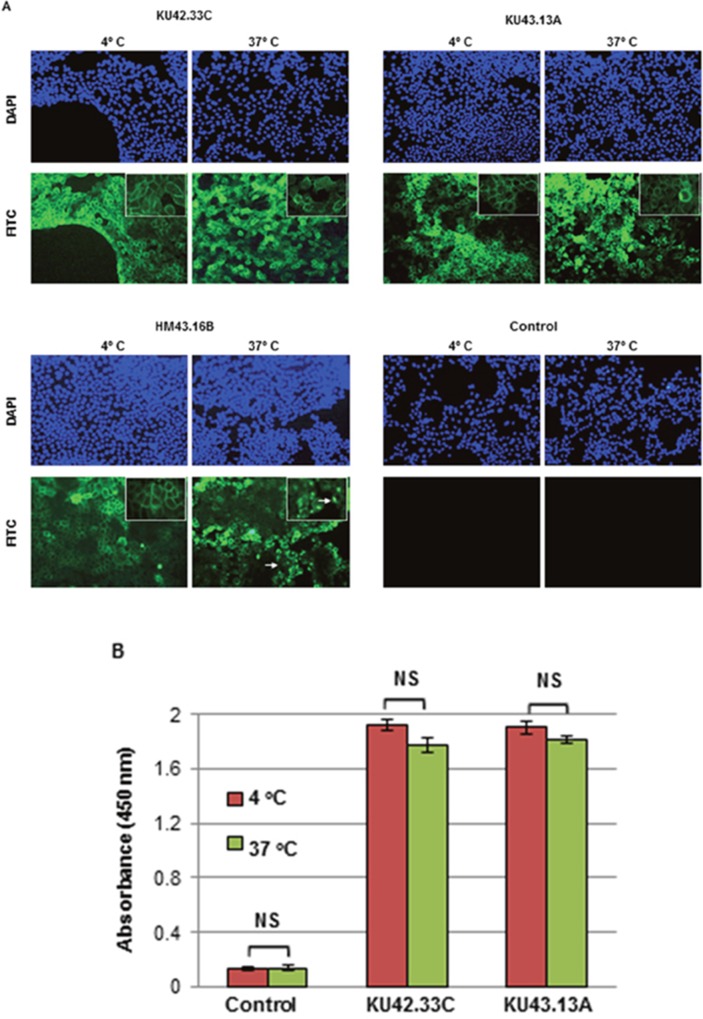
Internalisation studies of novel mAbs KU42.33C and KU43.13A in BxPC-3 human pancreatic cancer cells determined by **(A)** immunofluorescence, and **(B)** ELISA. BxPC-3 cancer cells were grown to near confluency and incubated with purified antibodies (50 μg/ml) or control (PBS/1% BSA) at 4ºC for 1 h and subsequently at 37ºC for extra 30 min to allow internalisation. Cells were then fixed, permeabilised and incubated with FITC-conjugated or HRP-linked anti-mouse secondary antibodies for immunofluorescence staining (200x magnifications) and ELISA respectively. The anti-EGFR mAb HM43.16B. was used as a control (arrows: internalised antibody). ELISA results (B) are presented as mean absorbance ± SD.

Next, we examined the effect of treatment with the two novel antibodies on growth and migration, *in vitro*, of a panel of human pancreatic cancer cell lines. At maximum concentration of 300 nM, mAbs KU42.33C and KU43.13A did not affect the growth of the human pancreatic cancer cell lines nor inhibited the migration of BxPC-3, AsPC-1 and CFPAC-1 cells (data not shown). We also examined the cellular location of the target antigens recognised by the mAbs. As shown in Figure 4A, the CD109 antigen recognised by these novel mAbs is located on the surface of human pancreatic cancer cells. In addition, there was no evidence of CD109 downregulation and internalisation following antibody treatment for 30 min at 37°C (Figures 4A & 4B). The anti-EGFR mAb HM43.16B was used as a control and treatment with this antibody resulted in the EGFR downregulation and internalisation (Figure 4A).

### The application of mAb KU42.33C in immunohistochemical detection of CD109 in pancreatic cancer

**Figure 5:**
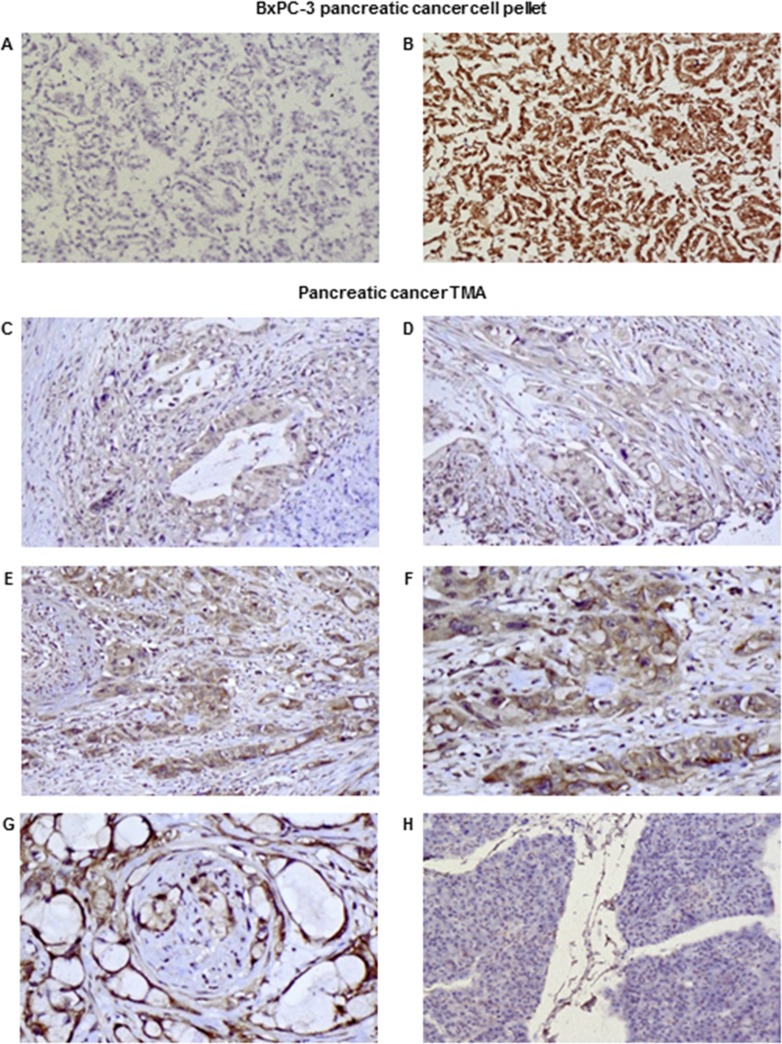
Examples of immunohistochemical staining of formalin-fixed, paraffin-embedded BxPC-3 cancer cell pellets **(A-B)** and pancreatic cancer tissue arrays **(C-H)** using novel mAbs KU43.13A (5 μg/ml; A) and KU42.33C (5 μg/ml; B-H). Negative and strong staining of BxPC-3 cell pellets with mAb KU43.13A **(A)** and mAb KU42.33C **(B)** respectively (magnification 200x). Representative images of staining of pancreatic cancer specimens in tissue arrays: **(C)** 1+ cytoplasmic staining, magnification 200x; **(D)** 1+ cytoplasmic/membranous staining, magnification 200x; **(E)** 2+ cytoplasmic/membranous staining, magnification 200x; **(F)** 2+ cytoplasmic/membranous staining, magnification 400x; **(G)** 2+ membranous staining with perineural invasion, magnification 400x; **(H)** Negative staining in normal pancreas, magnification 200x.

To explore the diagnostic potential of the novel mAbs, immunohistochemical staining of CD109 positive BxPC-3 tumour cell pellets was investigated. Our results showed that only mAb KU42.33C, which was found to be against a sequential epitope by Western blot analysis as described above, detected the antigen in formalin-fixed, paraffin-embedded tissue sections (Figure 5A and 5B). Further optimisation, including antigen retrieval with Tris-EDTA pH 9.0 buffer and signal amplification, resulted in stronger immunostaining of CD109 (data not shown). Next, we examined the relative expression of CD109 antigen by mAb KU42.33C in human pancreatic cancer tissue arrays containing 70 specimens from patients with pancreatic adenocarcinoma. Of these, five samples were either necrotic or contained no tumour and therefore were excluded from the study. Of 65 cases, 94% were CD109 positive, with intensity ranging from 1+ (n=55, 85%) to 2+ (n=6, 9%). Staining predominated in the membrane and the cytoplasm of cancer cells and did not correlate with the disease grade (Figure 5C-5G; Supplementary Table 1). In contrast, normal pancreatic tissue was negative or showed weak diffuse staining possibly attributable to technical artefact (Figure 5H). There was no staining in the absence of primary antibody-control (data not shown). Interestingly, with the exceptions of weak staining (1+ intensity) of normal tissues from ovary and small intestine, all the remaining 31 normal tissues from the following organs were CD109 negative: cerebellum, cerebral cortex, pituitary gland, spinal cord, eye, adrenal gland, thyroid, parathyroid adenoma, thymus, tonsil, bone marrow, kidney, bladder, prostate, testis, uterine cervix, endometrium, fallopian tube, oesophagus, stomach, colon, rectum, liver, pancreas, spleen, lung, heart, breast, placenta, striated muscle and skin (Supplementary Table 2).

## DISCUSSION

Pancreatic cancer is projected to become the second leading cause of cancer deaths in the USA by 2030 [7]. There is an urgent need to discover novel biomarkers of diagnostic, prognostic and predictive values and therapeutic targets in patients with pancreatic cancer. As mAb-based products are highly specific for their target antigens, they have been routinely used as diagnostic and therapeutic agents in many pathological conditions including cancer [9–12, 17–18]. Moreover, mAb technology is an excellent tool for the discovery of novel overexpressed cell surface tumour antigens and the study of their functions. Here, we report the generation of two mAbs, KU42.33C and KU43.13A, against two distinct epitopes on the external domain of CD109, using the human pancreatic cancer cell line BxPC-3 as the source of tumour immunogen. We have shown that the antigen recognised by these antibodies is overexpressed on the cell surface of BxPC-3 and other human pancreatic cancer cell lines by varying amounts (Figures 1 and 4A). However, treatment with these two antibodies did not affect the proliferation or migration *in vitro* of CD109 overexpressing cancer cell lines (data not shown), nor resulted in the down-regulation of CD109 antigen (Figure 4). Interestingly, of the two antibodies, only mAb KU42.33C recognised the CD109 antigen by Western blot (Figure 3B), which suggests that both mAbs are directed against different epitopes of the same antigen (Figure 2).

Originally identified by mAbs raised against the primitive CD34^+^ acute myeloid leukaemia cell line KG1a, CD109 is a transforming growth factor (TGF)-β co-receptor that binds TGF-β1, regulates TGF-β receptor endocytosis and degradation, and suppresses TGF-β/Smad signalling [19–23]. TGF-β has been found to play a role in growth, differentiation and migration; dysregulation of TGF-β signalling is associated with tissue fibrosis and cancer [21]. CD109 is a monomeric 170 KDa glycosylphosphatidylinositol (GPI)-anchored cell surface protein, a member of the α2-macroglobulin/C3, C4, C5 family of thioester-containing proteins [19, 21, 24]. CD109 is found on the cell surface of activated platelets and T-cells, endothelial cells and a subpopulation of CD34^+^ haematopoietic and progenitor cells, but is not expressed in most normal human tissues except for myoepithelial cells of mammary, lacrimal and salivary glands, and basal cells of bronchial and prostate epithelia [19, 20, 23, 25–27]. In addition to the 170 KDa major CD109 protein band, the identification of a 150 KDa band has been reported and found due to the proteolytic/autolytic cleavage of the 170 KDa counterpart [19, 25]. In agreement, we found that of our two novel antibodies, mAb KU42.33C, which targets a sequential epitope on the external domain of CD109, detected both the 170 KDa and 150 KDa protein bands by immunoprecipitation and Western blot (Figure 3A and 3B).

There is currently no comprehensive study on the expression pattern of CD109 determined by immunohistochemistry and its prognostic significance and predictive value for response to therapy in patients with pancreatic cancer. In only one study, Haun and colleagues examined the expression of CD109 in a panel of eight human pancreatic cancer cell lines by Western blot. They found high levels of expression in BxPC-3, MIA PaCa-2, and PANC-1 cells, with no/low expression in A818-4, AsPC-1, Capan-1, CFPAC-1 and Suit-2 cells [26]. These findings are consistent with the results obtained with our two anti-CD109 antibodies using ELISA and flow cytometry (Figure 1, Supplementary Figures 1 & 2). They also examined CD109 expression in 18 tissue sections from pancreatic ductal adenocarcinoma (PDAC) and 11 normal pancreatic tissue samples and found positive IHC CD109 staining of variable intensity in pancreatic carcinoma cells and completely negative or rare cases of focal and weak immunoreactivity in normal pancreatic tissue. A substantial difference in CD109 expression in pancreatic adenocarcinoma compared to normal pancreatic tissue was also observed and no staining was seen in other pancreatic tissue components such as blood vessels, pancreatic acini, stromal fibrous tissue, adipose tissue and inflammatory cells [26]. In our study, we examined pancreatic cancer tissue arrays of samples from 65 patients with different tumour grades and 94% of the cases were CD109-positive; we found no correlation between the staining intensity and tumour grade. The staining predominated in the cytoplasm of cancer cells although there some cases of coexisting membrane staining (Figures 5C-5G; Supplementary Table 1). Interestingly, the antibody did not stain normal human pancreatic tissue (Figure 5H). No staining was seen in a 31 out of 33 normal organs in tissue arrays (Supplementary Table 2).

Interestingly, CD109 has been found overexpressed in a number of other cancer types including carcinoma of the uterine cervix [28], lung squamous cell carcinoma [20], cancer of the oral cavity [29], breast cancer [30–31], malignant melanoma [32], myxofibrosarcoma [23], esophageal squamous cell carcinoma [22], squamous cell/adenosquamous carcinomas of the gallbladder [33], hepatocellular carcinoma [34], nasopharyngeal carcinoma [35] and penile squamous cell carcinoma [36]. For example, high levels of CD109 expression were observed by immunohistochemistry in squamous cell carcinomas and premalignant lesions of the oral cavity but not in normal tissue, suggesting that CD109 is useful in predicting transformation of premalignant lesions into cancer [29]. CD109 expression was also found to be more frequent in lung squamous cell carcinomas compared with other types of lung carcinoma including adenocarcinomas, large cell carcinomas and small cell carcinomas; however, no association between CD109 expression and the clinical stage of the disease was found [20]. Similarly, Dong and co-workers showed that CD109 is expressed in bladder squamous cell carcinomas and adenosquamous carcinomas but not in adenocarcinomas or normal gallbladder tissue [33]. A positive correlation has been reported between CD109 expression and tumour grade in patients with vulvar squamous cell carcinoma [37]. More recently, CD109 was found highly expressed in penile squamous cell carcinoma and to be a key regulator of the progression of lower-grade glioma and therefore a potential molecular target for therapy [36, 38].

There is currently conflicting data on the prognostic significance CD109 expression in such cancers. For example, CD109 expression determined by immunohistochemistry in tumour specimens from patients with myxofibrosarcoma and hepatocellular carcinoma was associated with a poorer prognosis in such patients [23, 34]. The expression of CD109 has also been reported in cancer stem-like cells/cancer-initiating cells (CSCs/CICs) in the epithelioid sarcoma cell line ESX, and was found to be associated with poor prognosis in patients with soft tissue sarcoma [39]. Additionally, a significant association was observed between high CD109 expression and low 1-year survival in patients with diffuse large B-cell lymphoma [40]. In contrast, CD109 expression was associated with better prognosis in patients with urothelial carcinomas [27]. CD109 expression was also found to be greater in triple-negative breast cancer (TNBC) compared to non-TNBC and to be associated with higher rate of distant metastasis and worse postoperative disease-specific survival compared to those with low or no expression [31]. In agreement, we also found CD109 overexpression in the triple negative EGFR overexpressing breast cancer cell line MDA-MB-468, but not in the ER-, PR-, HER2 overexpressing cell line SKBR-3 (Figure 1; Supplementary Figure 2). We also found overexpression of CD109 in other EGFR overexpressing head and neck squamous cell carcinoma cell line HN5 (Figure 1; Supplementary Figure 2). More recently, CD109 was reported to act as a novel pro-metastatic factor in a lung adenocarcinoma mouse model, and high levels of CD109 resulted in the activation of Jak-Stat3 signalling pathways in cancer cells suggesting that the direct targeting of CD109 could be of therapeutic benefit in the neoadjuvant or adjuvant setting [41]. While the soluble recombinant form of CD109 was reported to be capable of binding to TGF-β and antagonising TGF-β signalling and cellular responses in experimental system [42, 43], it remains unclear whether patients with pancreatic cancer or any other cancer type shed CD109 antigen into their sera and any other body fluids and if so, whether CD109 may confer diagnostic, prognostic and predictive values, and therefore warrants further investigation. Finally, while TMAs are commonly employed in medical research, the heterogeneous nature of both tumour and stroma in patients with pancreatic cancer support the need for further investigations on the relative expression and prognostic significance of CD109 in a larger group of patients using the whole tumour sections or several TMA cores from the same tumours [44, 45]

In summary, our results suggest that high expression of CD109 is common in pancreatic cancer. We believe that our two novel anti-CD109 mAbs, which are directed against two distinct epitopes on the extracellular domain of CD109, are ideal tools for conducting detailed studies of the biological significance and diagnostic, prognostic and predictive values of CD109 in human cancers. To our knowledge, there is currently no study on the importance of CD109 as target for therapy with mAbs in pancreatic cancer or any other type of cancer. Moreover, at present, the great majority of FDA approved therapeutic antibodies against tumour associated antigens (e.g. anti-CD20, anti-CD30, anti-EGFR, anti-HER-2 mAbs) are the humanised/chimeric IgG1 version of the mouse mAbs due to their superior ADCC (antibody-dependent cell-mediated cytotoxicity) and/or CDC (complement-dependent cytotoxicity) functions, or conjugated to toxins and radioactive substances to deliver lethal doses of such agents to the tumour. A major reason being that, with the exception of growth factor receptor blocking antibodies (e.g. anti-EGFR mAbs), treatment with the majority of naked mAbs do not inhibit the growth *in vitro* and migration of tumours overexpressing the target antigens [9, 17–18, 46]. As treatment with our two anti-CD109 mAbs did not affect the proliferation or migration of CD109 overexpressing cancer cell lines, further investigations are warranted to determine the therapeutic potential of the humanised IgG1 or the conjugated versions of such antibodies, for use in targeted therapy of tumours with CD109 overexpression.

## MATERIALS AND METHODS

### Cancer cell lines and cell culture

A panel of ten human pancreatic cancer cell lines was used in this study: BxPC-3, Capan-2, MIA PaCa-2, PANC-1, AsPC-1, HPAF-II, CFPAC-1, Capan-1, Hs766T and FA-6 [47]. BxPC-3, PANC-1, MIA PaCa-2 and SP2 myeloma cells were purchased from European Collection of Cell Cultures (ECACC, UK). AsPC-1, Capan-2, HPAF-II, Hs766T and CFPAC-1 were purchased from American Type Culture Collection (ATCC, UK). Capan-1 was kindly provided by Dr Charlotte Edling (Barts and The London School of Medicine and Dentistry) [47]. The EGFR overexpressing head and neck squamous cell carcinoma (HN5) and the breast carcinoma cell lines MDA-MB-468 and the HER-2 overexpressing SKBR-3 were also used as described previously [48]. MIA PaCa-2, PANC-1, Capan-1, MDA-MB468 and SKBR3 cells were authenticated by LGC Standards (Teddington, UK).

All cancer cell lines were cultured routinely at 37°C in a humidified atmosphere with 5% CO2. BxPC-3, AsPC-1, Capan-1 and FA-6 were cultured in RPMI-1640 medium, MIA PaCa-2, PANC-1 and Hs 766T in Dulbecco's Modified Eagle's Medium, HPAF-II in Eagle's Minimum Essential Medium, CFPAC-1 in Iscove's Modified Dulbecco's Medium and Capan-2 in McCoy's 5a Medium Modified. Cells were supplemented with 10% Foetal Bovine Serum (FBS) and antibiotics penicillin (50 units/ml), streptomycin (0.05 mg/ml) and neomycin (0.1 mg/ml). RPMI-1640 medium, Eagle's Minimum Essential Medium and Iscove's Modified Dulbecco's Medium were supplemented with 2 mM, 4 mM and 8 mM L-Glutamine respectively. MDA-MB-468, SKBR-3, HN5 and SP2 cells were grown in Dulbecco's Modified Eagle's Medium supplemented with 10% FBS and antibiotics. All culture media and additives were purchased from Sigma Aldrich, UK.

### Generation of novel monoclonal antibodies

The mice immunisation was performed at St George's University of London, following the ethical approval and under Home Office animal license. Female BALB/c mice (aged 5-6 weeks) were immunised repeatedly with subcutaneous flank injections plus an intraperitoneal (i.p.) injection of BxPC-3 human pancreatic cancer cells (3 sites; 100 μl per site) with a total number of 10 million tumour cells per immunisation per mouse. Immunisation was repeated 2 times every 2 weeks and the final injection was administered 3-4 days before collection of lymphocytes from the spleen of immunised mice. Spleens were removed from the immunised animals by blunt dissection, disaggregated, and the cells washed, centrifuged and resuspended in a freezing down solution for storage in liquid nitrogen until the date of fusion.

B-lymphocytes derived from the spleen of immunised mice were fused with SP2 myeloma cells, facilitated by 50% polyethylene glycol (PEG; Sigma Aldrich). Cells were cultured in HAT medium (Sigma Aldrich) supplemented with 20% FBS, 10% Hybridoma Cloning Supplement (Santa Cruz Biotechnology, UK) and antibiotics. Newly formed hybridomas were screened by ELISA, cloned twice by limiting dilution technique, grown in roller bottles and the hybridoma supernatants were harvested. Isotyping of novel mAbs was determined using a mouse mAb isotyping kit (AbD Serotec, UK) according to the manufacturer's protocol and the antibodies were purified by affinity chromatography (below).

### Antibody screening by ELISA and flow cytometry analysis

Antibodies secreted by novel hybridomas were screened by ELISA. Briefly, cancer cells were seeded in 96-well plates and incubated until near confluent. Cells were washed once and then incubated with novel hybridoma supernatants or purified mAbs (25 μg/ml) on ice for 1 h, followed by incubation with 50 μl HRP-conjugated rabbit anti-mouse secondary antibody (1:1000, STAR13B, AbD Serotec) on ice for 45 min. Cells were washed and then 50 μl TMB (3,3',5,5'-Tetramethylbenzidine; Sigma Aldrich) added to each well and left for ∼15 min at room temperature; the TMB reaction was stopped by adding 50 μl of 0.5 M sulphuric acid (H_2_SO_4_) to each well. Positive and negative controls were included. The absorbance of each sample was measured at 450 nm using a Biotek plate reader (Biotek, UK).

The cell surface expression of target antigens recognised by novel mAbs was determined using flow cytometry as described previously [49]. Briefly, cells were dissociated from the tissue culture flasks using enzyme-free cell dissociation buffer (Fisher Scientific, UK). Approximately 1×10^6^ tumour cells were incubated with novel mouse mAbs KU42.33C or KU43.13A (10 μg/ml) or control (i.e. PBS) by rotation for 1 h at 4°C, followed by incubation with FITC-conjugated goat anti-mouse IgG secondary antibody (1:200; STAR9B, AbD Serotec) for 45 min at 4°C. A minimum of 10,000 events were recorded by excitation with an argon laser at 488 nm and analysed using the FL-1 detector (FITC detector; 525 nm) of a BD FACScalibur flow cytometer using CellQuest Pro software (Becton-Dickinson Ltd, UK).

### Purification of novel monoclonal antibodies

The purification of mAbs was performed by affinity chromatography. Briefly, novel mouse mAbs were purified by salt fractionation (solid ammonium sulphate ([NH_4_]_2_SO_4_; 45% of saturation - 270 g/L; Fisher Scientific) followed by affinity chromatography using a 5 ml HiTrap Protein G HP column in an ÄKTAprime plus chromatography system (both from GE Healthcare, UK). The antibody sample was loaded into the HiTrap Protein G HP column pre-equilibrated with 25 ml binding buffer (0.1 M phosphate buffer, 0.15 M NaCl, pH 7.4) at a flow rate of 1 ml/min. Unbound proteins were removed by washing the column with 50 ml binding buffer and bound antibodies were eluted with 25 ml elution buffer (0.1 M glycine, pH 2.5). Selected fractions were pooled together and subjected to buffer exchange with PBS using the HiPrep 26/10 Desalting column (GE Healthcare Life Sciences). Following buffer exchange, the purified antibodies were filtered through a 0.2 μM syringe filter (Merck Millipore, UK), aliquoted and stored at -20°C for further studies.

### Competitive binding assay

BxPC-3 cancer cells were seeded in 96-well plates and incubated until they became near confluent. Cells were washed once with serum-free DMEM and increasing amounts of mAb KU42.33C were added to different wells in duplicates until near saturation point. Thereafter, increasing concentrations of either mAb KU42.33C or KU43.13A were added in duplicates and incubated on ice for 1 hour. Following washing, 50 μl HRP-conjugated rabbit anti-mouse secondary antibody (1:1000, STAR13B, AbD Serotec) was added and incubated on ice for 45 min. Cells were washed again three times and 50 μl TMB added to each well and left for ∼15 min at room temperature; the TMB reaction was stopped by adding 50 μl of 0.5 M sulphuric acid (H_2_SO_4_) to each sample and its absorbance measured at 450 nm.

### Internalisation studies

Immunofluorescence staining of tumour cells and ELISA were used to determine whether treatment with novel antibodies resulted in down-regulation of the target antigen. Briefly, BxPC-3 cancer cells were grown to near confluency in RPMI/10% FBS in Lab-Tek 8-well chamber slides (VWR, UK) or 96-well plates, respectively. Cells were incubated with mAbs KU42.33C or KU43.13A (50 μg/ml), or negative control (i.e. PBS/1%BSA alone) for 1 h at 4°C to allow antibody binding, followed by incubation at 37°C for 30 min to allow antibody internalisation. The anti-EGFR mAb HM43.16B was used as another control in this study [50]. A control slide was maintained at 4°C. Cells were fixed with 4% formaldehyde for 10 min, the cell membrane permeabilised with 0.5% Triton-X 100 for 15 min and non-specific binding blocked with PBS/1%BSA for 1 h at 4°C. Cells in immunofluorescence slides were then incubated with Alexa Fluor 488 secondary antibody (1:200; Fisher Scientific) for 1 h at 4°C, mounted in Vectashield with DAPI (Vector laboratories, UK) and examined using Nikon eclipse i80 microscope and Nikon NIS-Elements software as described previously [51]. Cells in ELISA plates were incubated with HRP-conjugated rabbit anti-mouse secondary antibody and the absorbance of each sample measured at 450 nm.

### Cell growth studies

The effect of novel mAbs on the growth of human cancer cell lines was investigated using the Sulforhodamine B (SRB) colorimetric assay [47, 49]. Approximately 5×10^3^ tumour cells per well were seeded in 96-well plates containing medium supplemented with 2% FBS in the presence of mAbs (300 nM) or control medium. AsPC-1 and HPAF-II cancer cells were grown in 5% FBS medium as they did not grow well in 2% FBS medium. A (no treatment) control plate was included under the same conditions (4 h incubation at 37°C) to determine the initial number of cells before treatment. Cells were cultured until the controls became almost confluent (4-10 days). Tumour cells were fixed with 10% trichloroacetic acid (TCA; Fisher Scientific), stained with 0.4% SRB (Sigma Aldrich) in 1% acetic acid and solubilised with Tris-base (10 mM, pH 10; Fisher Scientific) before plate reading. Gen5 software was used to determine the IC50 through non-linear least squares curve fitting [47, 49].

### Migration assay

The effect of novel mAbs on the migration of human pancreatic cancer cells BxPC-3, AsPC-1 and CFPAC-1 was investigated using the IncuCyte ZOOM® Live-Cell Imaging instrument (Essen Bioscience, UK) as described previously [52]. Approximately 1×10^3^ cancer cells per well were seeded in duplicates in 0.5% FBS medium in an IncuCyte™ ClearView 96-well Cell Migration Plate (Essen Bioscience, UK) along with 300 nM mAbs or control (i.e. medium alone). The cells were allowed to settle for 15 min at room temperature before transfer to an incubator at 37°C for 30 min to pre-incubate the cells in the presence of treatment. Then, 200 μl of chemoattractant (i.e. 10% FBS medium) or control (i.e. 0.5% FBS medium) were added to the appropriate wells of the reservoir plate and the insert plate placed into the pre-filled reservoir plate. The plate was then transferred to the IncuCyte ZOOM® Live-Cell Imaging Instrument (Essen Bioscience) and allowed to warm to 37°C for 15 min before any condensation accumulated on the plate lid or bottom was wiped away. The plate was imaged at 10x objective using the Chemotaxis Scan Type - Phase channel. The IncuCyte™ Chemotaxis Cell Migration Software Module (Essen Bioscience) was used for data analysis. Whole-well images of cells on both the bottom and the top of the plate membrane were captured every 2 h over 48 h and all images were processed using automatic algorithms to quantify cell area on each side of the membrane.

### Immunoprecipitation and mass spectrometry

To identify the target antigens recognised by the antibodies, immunoprecipitation and mass spectrometry were performed. Briefly, novel mAbs (5 μg) were incubated with 1 ml BxPC-3 tumour cell lysates (lysis buffer containing 50 mM Tris-HCl pH 7.2, 150 mM NaCl, 2 mM MgCl_2_, 2 mM CaCl_2_, 0.1% NaN_3_, 100 mM DTT, 1% Triton X-100, 50 mM N-ethylmaleimide) overnight at 4°C by gentle rotation (14 rpm), and then incubated with 50 μl pre-washed Dynabeads sheep anti-mouse IgG for 1 h at 4°C. The immunocomplexes were captured on a DynaMag™-2 for 2 min, the supernatants aspirated and the samples washed 3 times with PBS. The complexes were then eluted by mixing beads with LDS sample buffer (25% NuPAGE LDS buffer [4x], 10% reducing agent [10x] and 65% distilled water; Invitrogen, UK), heated to 95°C for 5 min and analysed by SDS-PAGE.

Identification of isolated protein was performed by mass spectrometry at the University of York. Desired bands were excised and in-gel digested with trypsin. Positive-ion MALDI mass spectra were obtained using a Bruker ultraflex III in reflectron mode, equipped with a Nd:YAG smart beam laser. MS spectra were acquired over a mass range of m/z 800-5000. Final mass spectra were externally calibrated against an adjacent spot containing 6 peptides (des-Arg1-Bradykinin, 904.681; Angiotensin I, 1296.685; Glu1-Fibrinopeptide B, 1750.677; ACTH (1-17 clip), 2093.086; ACTH (18-39 clip), 2465.198; ACTH (7-38 clip), 3657.929). Monoisotopic masses were obtained using a SNAP averaging algorithm (C 4.9384, N 1.3577, O 1.4773, S 0.0417, H 7.7583) and a S/N threshold of 2. For each spot, the ten strongest peaks of interest, with a S/N greater than 30, were selected for MS/MS fragmentation. Fragmentation was performed in LIFT mode without the introduction of a collision gas. The default calibration was used for MS/MS spectra, which were baseline-subtracted and smoothed (Savitsky-Golay, width 0.15 m/z, cycles 4); monoisotopic peak detection used a SNAP averaging algorithm (C 4.9384, N 1.3577, O 1.4773, S 0.0417, H 7.7583) with a minimum S/N of 6. Bruker flexAnalysis software (version 3.3) was used to perform the spectral processing and peak list generation for both the MS and MS/MS spectra. Tandem mass spectral data were submitted to database searching using a locally-running copy of the Mascot program (Matrix Science Ltd., version 2.1), through the Bruker BioTools interface (version 3.2). Search criteria included: Enzyme, Trypsin; Fixed modifications, Carbamidomethyl (C); Variable modifications, Oxidation (M); Peptide tolerance, 100 ppm; MS/MS tolerance, 0.5 Da; Instrument, MALDI-TOF-TOF. UniProt_human_SP database was used for protein identification.

### Western blotting

The ability of the novel antibodies to recognise the target antigen in Western blot was performed. Briefly, protein immunoprecipitated with novel mAbs KU42.33C and KU43.13A (5 μg) was analysed by SDS-PAGE under reducing conditions, prior to Western blotting. The transfer of proteins from 4-12% Bis-Tris-gels to Immobilon-FL PVDF membranes (Merck Millipore, UK) was performed using the XCell II™ Mini-Cell Blot Module kit (Invitrogen, UK) at a constant voltage of 30 V on ice for 2 h. PVDF membranes were probed with novel mAbs (30 μg/ml) or commercial anti-CD109 mAb (1:100; sc-365780; Santa Cruz Biotechnology, UK) overnight at 4°C and subsequently incubated with secondary goat anti-mouse antibody (1:10,000; LI-COR Biosciences, UK) for 1 h at room temperature. For visualisation, the blots were analysed using the Oddysey® CLx instrument (LI-COR Biosciences).

### Immunohistochemical examination of tumours

BxPC-3 cell pellets were used to determine whether novel antibodies can be used for detection of the target antigen in formalin-fixed paraffin-embedded tissues and for further optimisation of the primary antibodies. Then, the expression level of the target antigens was determined in human pancreatic cancer tissue arrays (35 cases in early stage, 35 cases in advance stage and 10 cases normal tissue; Cat. No. PA804a, Biomax US) and normal organ tissue arrays (33 types of normal tissue; Cat. No. MNO661, Biomax US) by the mAb. Briefly, tissue sections were deparaffinised and rehydrated through a series of alcohols followed by antigen retrieval with Tris-EDTA buffer (10 mM TrisBase, 1 mM EDTA, 0.05% Tween 20, pH 9.0). Then, the mouse-specific HRP/DAB (ABC) Detection IHC Kit (Cat. No. ab64259, Abcam, UK) was used according to the manufacturer instructions. Briefly, tissue sections were incubated with hydrogen peroxide block for 10 min, followed by protein block for 5 min and subsequent incubation with novel mAbs (5 μg/ml) overnight at 4°C. Following washing, the tissue sections were incubated with biotinylated goat anti-mouse secondary antibody for 10 min, followed by streptavidin peroxidase for another 10 min. After washing, the colorimetric reaction was developed by incubation with DAB substrate plus chromogen for 5 min. The slides were counterstained with Harris Haematoxylin (VWR, UK) for 2 min and rinsed with water. The tissue sections were then dehydrated through a series of alcohols and the slides mounted in DPX mounting medium (VWR) and examined for the expression level and intensity of staining (i.e. 1+ weak, 2+ moderate, 3+ strong).

### Statistical analysis

Statistical analysis was performed using the Statistical Package for the Social Sciences (SPSS) version 23 (SPSS UK Ltd, Woking, UK). The unpaired two-tailed Student's t-test was used to compare mean values between two groups. Data are presented as mean ± SD. P ≤ 0.05 was considered statistically significant.

## SUPPLEMENTARY MATERIALS FIGURES AND TABLES





## Abbreviations

ADCC: antibody-dependent cell-mediated cytotoxicity
CDC: complement-dependent cytotoxicity
CICs: cancer-initiating cells
CSCs: cancer stem-like cells
GPI: glycosylphosphatidylinositol
IHC: immunohistochemistry
mAbs: monoclonal antibodies
MFI: mean fluorescence intensity
SRB: Sulforhodamine B
TGF: transforming growth factor
TNBC: triple-negative breast cancer
