# Supplementary material for: Development of novel monoclonal antibodies against CD109 overexpressed in human pancreatic cancer
Source: Oncotarget. 2018 Apr 13;9(28):19994–20007. doi: 10.18632/oncotarget.25017 (PMC5929441; doi:10.18632/oncotarget.25017)
Supplement: Supplementary file 1 [file oncotarget-09-19994-s001.pdf]

Development of novel monoclonal antibodies against CD109 overexpressed in human pancreatic cancer

SUPPLEMENTARY MATERIALS

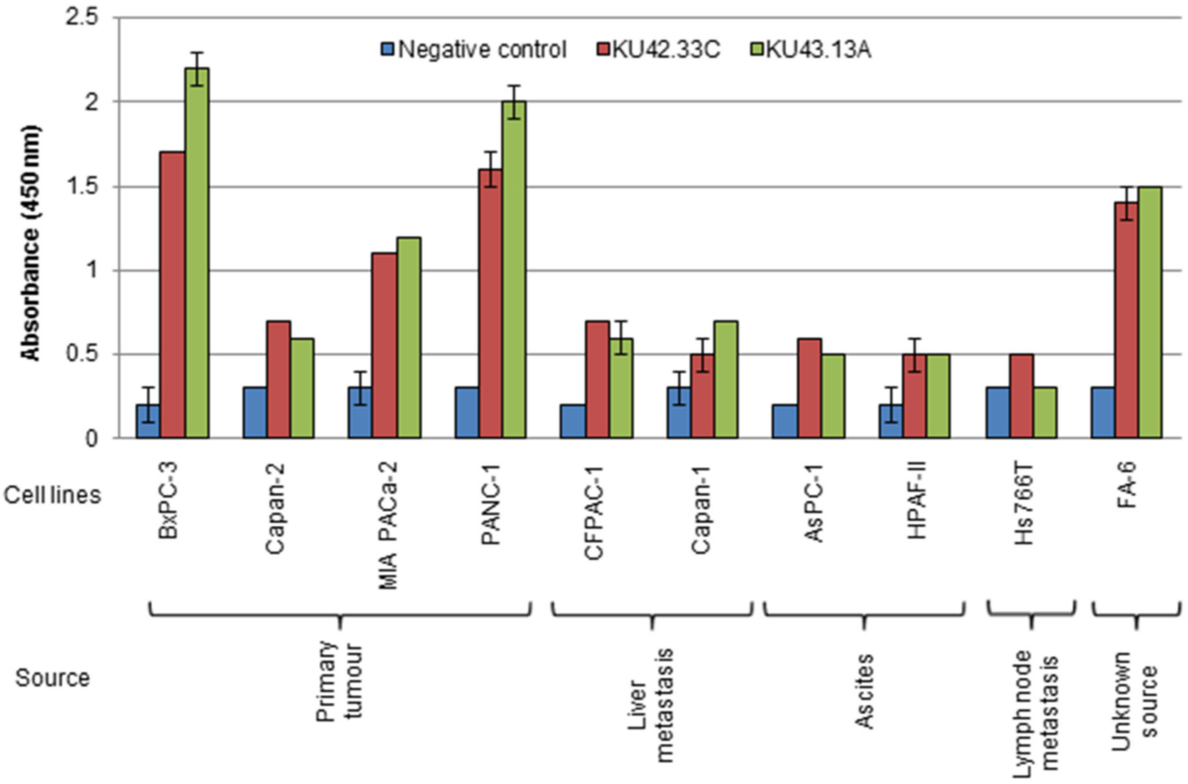

Supplementary Figure 1: Expression level of the antigens recognised by novel mAbs KU42.33C and KU43.13A in a panel of human pancreatic cancer cell lines determined by ELISA. Results are presented as mean absorbance ± SD.

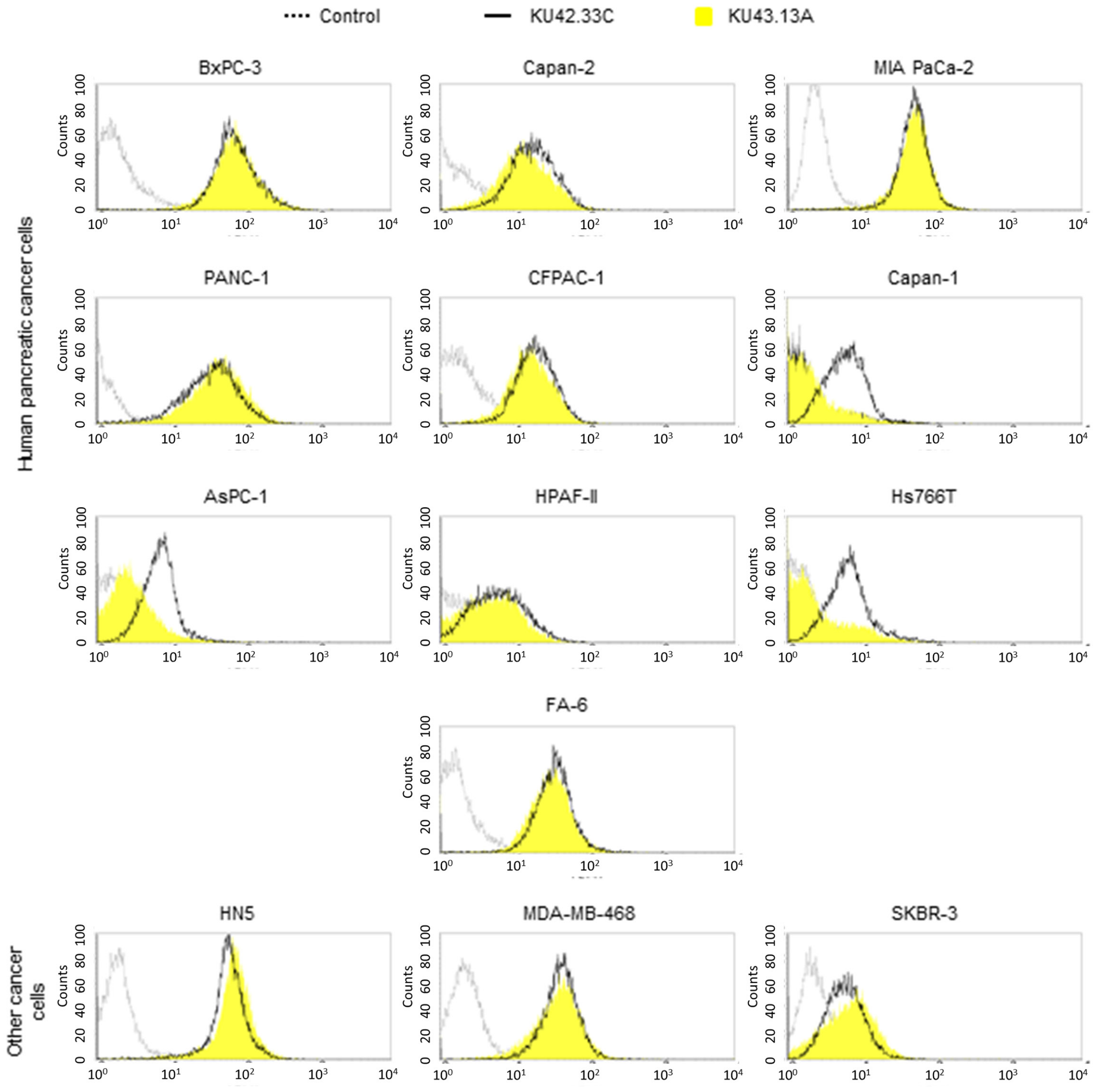

Supplementary Figure 2: Expression level of the antigens recognised by novel mAbs KU42.33C and KU43.13A on human pancreatic cancer and other cancer cell lines, presented as histogram.

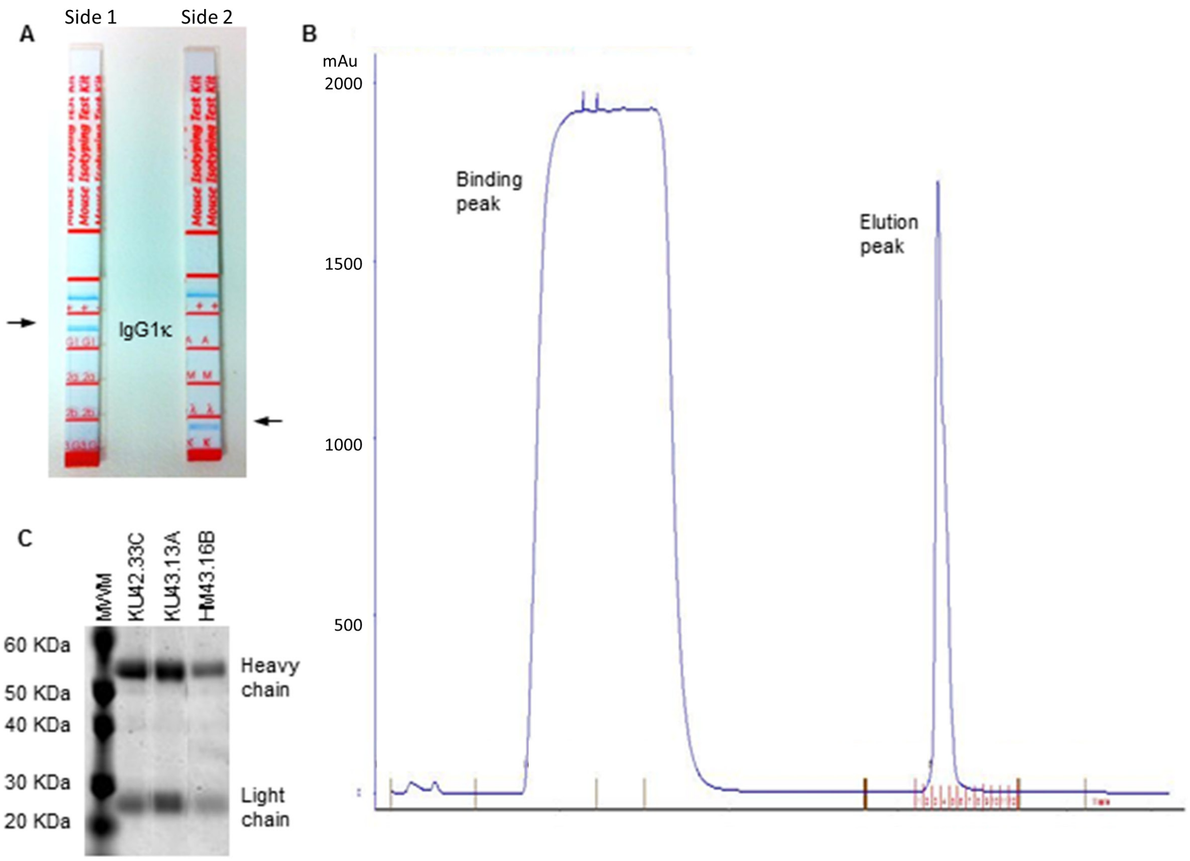

Supplementary Figure 3: Purification of novel monoclonal antibodies. (A) The isotype of mAb KU43.13A determined by mouse mAb Isotyping kit (AbD, Serotec). Top blue band represent the positive control. **(B)** Example of purification of mAb KU43.13A determined by HiTrap Protein G HP column in an ÄKTAprime plus chromatography system (both from GE Healthcare, UK). **(C)** Coomassie blue staining of purified monoclonal antibodies analysed by SDS-PAGE under reducing conditions. HM43.16B is a control mouse anti-EGFR IgG1 mAb. MWM: molecular weight marker.

Supplementary Table 1: Patient characteristics, disease grade and staining intensity and distribution of pancreatic cancer samples in tissue microarray (Cat No. PA804a, Biomax US)

See Supplementary File 1

Supplementary Table 2: Patient characteristics and anatomic site of normal tissue microarray (Cat No. MNO661, Biomax US)

No.	Age	Sex	Anatomic site	Scoring
1	37	F	Adrenal gland	negative
2	53	M	Bladder	negative
3	16	M	Bone marrow	negative
4	45	F	Eye	negative
5	46	F	Breast	negative
6	58	M	Cerebellum	negative
7	58	M	Cerebral cortex	negative
8	38	F	Fallopian tube	negative
9	66	M	Esophagus	negative
10	53	F	Stomach	negative
11	47	M	Small intestine	1+
12	26	M	Colon	negative
13	83	M	Rectum	negative
14	58	M	Heart	negative
15	62	M	Kidney	negative
16	67	F	Liver	negative
17	45	F	Lung	negative
18	51	F	Ovary	1+
19	35	M	Pancreas	negative
20	25	M	Parathyroid (adenoma)	negative
21	58	M	Pituitary gland	negative
22	38	F	Placenta	negative
23	61	M	Prostate	negative
24	58	M	Skin	negative
25	1	F	Spinal cord	negative
26	70	M	Spleen	negative
27	46	M	Striated muscle	negative
28	70	M	Testis	negative
29	15	F	Thymus	negative
30	29	F	Thyroid	negative
31	6	F	Tonsil	negative
32	47	F	Uterine cervix	negative
33	41	F	Endometrium	negative
