# Supplementary material for: Development of novel monoclonal antibodies against CD109 overexpressed in human pancreatic cancer
Source: Oncotarget. 2018 Apr 13;9(28):19994–20007. doi: 10.18632/oncotarget.25017 (PMC5929441; doi:10.18632/oncotarget.25017)
Supplement: Supplementary file 2 [file oncotarget-09-19994-s002.docx]

**Supplementary Table 1. Patient characteristics, disease grade and staining intensity and distribution of pancreatic cancer samples in tissue microarray (Cat No. PA804a, Biomax US).**

| **No.** | **Age** | **Sex** | **Pathology diagnosis** | **TNM** | **Grade** | **Scoring** |
| --- | --- | --- | --- | --- | --- | --- |
| 1 | 52 | M | Duct adenocarcinoma | T1N0M0 | 1 | 1+ c |
| 2 | 77 | M | Duct adenocarcinoma | T1N0M0 | 2 | 1+ c |
| 3 | 56 | F | Duct adenocarcinoma | T2N0M0 | 1 | 1+ c |
| 4 | 80 | M | Duct adenocarcinoma | T2N0M0 | 1 | 1+ c |
| 5 | 54 | F | Duct adenocarcinoma | T2N0M0 | 1 | no tumour |
| 6 | 47 | M | Duct adenocarcinoma | T2N0M0 | 1 | 1+ c |
| 7 | 54 | M | Duct adenocarcinoma | T2N0M0 | 2 | 1+ c |
| 8 | 41 | M | Mucinous adenocarcinoma | T2N0M0 | 2 | 1+ c/m |
| 9 | 56 | M | Duct adenocarcinoma | T2N0M0 | 1 | 1+ c/m |
| 10 | 70 | M | Duct adenocarcinoma | T2N0M0 | 1-2 | no tumour |
| 11 | 62 | M | Duct adenocarcinoma | T2N0M0 | 3 | 1+ c/m |
| 12 | 44 | F | Adenocarcinoma | T2N0M0 | 2 | necrosis |
| 13 | 54 | F | Adenocarcinoma | T2N0M0 | 2 | 1+ c |
| 14 | 50 | M | Adenocarcinoma | T2N0M0 | 2 | 1+ c/m |
| 15 | 68 | F | Duct adenocarcinoma | T2N0M0 | 2 | 2+ c/m |
| 16 | 65 | M | Mucinous adenocarcinoma | T2N0M0 | 3 | 1+ c |
| 17 | 58 | F | Adenocarcinoma | T2N0M0 | 2 | 1+ c/m |
| 18 | 52 | M | Adenocarcinoma (sparse) | T2N0M0 | 2-3 | 1+ c |
| 19 | 54 | F | Duct adenocarcinoma | T2N0M0 | 3 | 1+ c |
| 20 | 60 | M | Adenocarcinoma | T2N0M0 | 2-3 | 1+ c |
| 21 | 45 | M | Adenocarcinoma | T2N0M0 | 2-3 | no tumour |
| 22 | 63 | M | Adenocarcinoma | T2N0M0 | 3 | 1+ c |
| 23 | 59 | M | Adenocarcinoma | T2N0M0 | 2 | 1+ c |
| 24 | 65 | M | Adenocarcinoma | T2N0M0 | 3 | 1+ c |
| 25 | 23 | F | Adenocarcinoma | T2N0M0 | 3 | negative |
| 26 | 51 | F | Adenocarcinoma | T2N0M0 | 3 | 1+ c |
| 27 | 53 | M | Adenocarcinoma | T2N0M0 | 2 | 1+ c |
| 28 | 55 | M | Adenocarcinoma with necrosis | T2N0M0 | 2 | 1+ c |
| 29 | 57 | F | Adenocarcinoma | T2N0M0 | 3 | 1+ c/m |
| 30 | 58 | F | Adenocarcinoma | T1N0M0 | 2 | 1+ c/m |
| 31 | 56 | M | Adenocarcinoma | T2N0M0 | 3 | 1+ c/m |
| 32 | 56 | M | Adenocarcinoma | T2N0M0 | 3 | 1+ c |
| 33 | 55 | M | Adenocarcinoma | T2N0M0 | 3 | negative |
| 34 | 40 | F | Islet cell carcinoma | T2N0M0 | - | 1+ c |
| 35 | 37 | F | Neuroendocrine carcinoma | T2N0M0 | - | negative |
| 36 | 62 | F | Adenocarcinoma | T3N0M0 | 1 | 1+ c/m |
| 37 | 72 | F | Duct adenocarcinoma | T3N0M0 | 1 | 1+ c |
| 38 | 48 | F | Duct adenocarcinoma | T3N0M0 | 1 | 1+ c/m |
| 39 | 60 | M | Adenocarcinoma | T2N1M0 | 3 | 1+ c/m |
| 40 | 47 | F | Adenocarcinoma (sparse) | T3N0M0 | 1 | 1+ c |
| 41 | 34 | M | Duct adenocarcinoma | T3N0M0 | 1 | 2+ c |
| 42 | 51 | F | Adenocarcinoma | T3N0M0 | 1-2 | 1+ c/m |
| 43 | 68 | M | Mucinous adenocarcinoma | T2N1M0 | 3 | 2+ c |
| 44 | 39 | M | Duct adenocarcinoma | T3N0M0 | 3 | 1+ c/m |
| 45 | 42 | F | Adenocarcinoma | T3N0M0 | 2 | 1+ c/m |
| 46 | 54 | M | Duct adenocarcinoma | T3N0M0 | 2 | 1+ c |
| 47 | 42 | M | Adenocarcinoma | T3N0M0 | 2 | 2+ c |
| 48 | 51 | M | Duct adenocarcinoma | T3N0M0 | 2 | 1+ c |
| 49 | 60 | M | Adenocarcinoma | T3N0M0 | 2 | 1+ c |
| 50 | 51 | F | Adenocarcinoma | T3N0M0 | 3 | 1+ c/m |
| 51 | 51 | M | Adenocarcinoma | T3N0M0 | 3 | 2+ c/m |
| 52 | 58 | F | Adenocarcinoma | T3N0M0 | 2-3 | 1+ c/m |
| 53 | 64 | M | Duct adenocarcinoma | T3N0M0 | 3 | 1+ c/m |
| 54 | 74 | M | Adenocarcinoma | T3N0M0 | 3 | 2+ c |
| 55 | 56 | M | Duct adenocarcinoma | T2N1M0 | 1 | 1+ c/m |
| 56 | 40 | F | Duct adenocarcinoma | T3N1M0 | 1 | 1+ c/m |
| 57 | 58 | F | Duct adenocarcinoma | T3N1M0 | 1 | 1+ c/m |
| 58 | 67 | F | Duct adenocarcinoma | T3N1M0 | 1 | 1+ c/m |
| 59 | 48 | F | Duct adenocarcinoma | T3N1M0 | 1 | 1+ c |
| 60 | 62 | F | Adenocarcinoma | T2N1M0 | 1-2 | 1+ c |
| 61 | 50 | M | Duct adenocarcinoma | T2N1M0 | 1-2 | negative |
| 62 | 40 | M | Duct adenocarcinoma | T2N1M0 | 2 | 1+ c |
| 63 | 49 | M | Adenocarcinoma | T2N1M0 | 2 | 2+ c |
| 64 | 62 | M | Adenocarcinoma | T3N1M0 | 2 | 1+ c/m |
| 65 | 49 | M | Duct adenocarcinoma | T3N1M0 | 3 | 1+ c |
| 66 | 65 | M | Adenocarcinoma | T3N1M0 | 2-3 | no tumour |
| 67 | 58 | F | Adenocarcinoma | T4N0M0 | 2 | 1+ c |
| 68 | 41 | M | Adenocarcinoma | T4N1M0 | 3 | 1+ c |
| 69 | 46 | F | Adenocarcinoma | T4N0M0 | 3 | 1+ c |
| 70 | 62 | M | Squamous cell carcinoma | T3N0M0 | 2-3 | 1+ c |
| 71 | 47 | M | Normal pancreatic tissue | - | - | negative |
| 72 | 35 | F | Normal pancreatic tissue | - | - | negative |
| 73 | 38 | F | Normal pancreatic tissue | - | - | negative |
| 74 | 35 | M | Normal pancreatic tissue | - | - | negative |
| 75 | 21 | F | Normal pancreatic tissue | - | - | negative |
| 76 | 21 | F | Normal pancreatic tissue | - | - | negative |
| 77 | 50 | M | Normal pancreatic tissue | - | - | negative |
| 78 | 21 | F | Normal pancreatic tissue | - | - | negative |
| 79 | 23 | M | Normal pancreatic tissue | - | - | negative |
| 80 | 40 | M | Normal pancreatic tissue | - | - | negative |

c: cytoplasmic; m: membranous
